# EARL compliance and imaging optimisation on the Biograph Vision Quadra PET/CT using phantom and clinical data

**DOI:** 10.1007/s00259-022-05919-1

**Published:** 2022-07-25

**Authors:** Joyce van Sluis, Johannes H. van Snick, Adrienne H. Brouwers, Walter Noordzij, Rudi A. J. O. Dierckx, Ronald J. H. Borra, Riemer H. J. A. Slart, Adriaan A. Lammertsma, Andor W. J. M. Glaudemans, Ronald Boellaard, Charalampos Tsoumpas

**Affiliations:** 1 Department of Nuclear Medicine and Molecular Imaging, University of Groningen, University Medical Center Groningen, Groningen, The Netherlands; 2 Department of Radiology and Nuclear Medicine, Amsterdam University Medical Centers, location VUMC, Amsterdam, The Netherlands

**Keywords:** Imaging optimization, EARL, Scan duration, SiPM, LAFOV PET/CT

## Abstract

**Purpose:**

Current European Association of Nuclear Medicine (EANM) Research Ltd. (EARL) guidelines for the standardisation of PET imaging developed for conventional systems have not yet been adjusted for long axial field-of-view (LAFOV) systems. In order to use the LAFOV Siemens Biograph Vision Quadra PET/CT (Siemens Healthineers, Knoxville, TN, USA) in multicentre research and harmonised clinical use, compliance to EARL specifications for ^18^F-FDG tumour imaging was explored in the current study. Additional tests at various locations throughout the LAFOV and the use of shorter scan durations were included. Furthermore, clinical data were collected to further explore and validate the effects of reducing scan duration on semi-quantitative PET image biomarker accuracy and precision when using EARL-compliant reconstruction settings.

**Methods:**

EARL compliance phantom measurements were performed using the NEMA image quality phantom both in the centre and at various locations throughout the LAFOV. PET data (maximum ring difference (MRD) = 85) were reconstructed using various reconstruction parameters and reprocessed to obtain images at shorter scan durations. Maximum, mean and peak activity concentration recovery coefficients (RC) were obtained for each sphere and compared to EARL standards specifications.

Additionally, PET data (MRD = 85) of 10 oncological patients were acquired and reconstructed using various reconstruction settings and reprocessed from 10 min listmode acquisition into shorter scan durations. Per dataset, SUVs were derived from tumour lesions and healthy tissues. ANOVA repeated measures were performed to explore differences in lesion SUV_max_ and SUV_peak_. Wilcoxon signed-rank tests were performed to evaluate differences in background SUV_peak_ and SUV_mean_ between scan durations. The coefficient of variation (COV) was calculated to characterise noise.

**Results:**

Phantom measurements showed EARL compliance for all positions throughout the LAFOV for all scan durations. Regarding patient data, EARL-compliant images showed no clinically meaningful significant differences in lesion SUV_max_ and SUV_peak_ or background SUV_mean_ and SUV_peak_ between scan durations. Here, COV only varied slightly.

**Conclusion:**

Images obtained using the Vision Quadra PET/CT comply with EARL specifications. Scan duration and/or activity administration can be reduced up to a factor tenfold without the interference of increased noise.

**Supplementary Information:**

The online version contains supplementary material available at 10.1007/s00259-022-05919-1.

## Introduction

The non-invasive imaging technique positron emission tomography (PET) integrated with computed tomography (CT) is widely used in oncology [1–3] and many other clinical indications, providing both metabolic and anatomic information [4]. In oncology, PET/CT is a rapidly evolving technique which has become part of the daily clinical routine for initial diagnosis, staging, radiation therapy planning, prognosis and treatment-response monitoring [3, 5, 6].

The most frequently used PET tracer in oncology is 2-deoxy-2-[fluorine-18] fluoro-D-glucose (^18^F-FDG) [7]. Acquired ^18^F-FDG images can be interpreted visually, e.g., for staging, or semi-quantitatively, e.g., to determine treatment-response, which requires standardised imaging procedures, especially in a multicentre setting [8]. When procedure guidelines for tumour imaging are followed, PET images can be converted to standardised uptake values (SUV), normalising the radioactive activity concentration as depicted in the image by body weight and amount of injected tracer activity. Using SUVs as a metric of relative tissue uptake facilitates comparisons between patients [7].

Recently, long axial field-of-view (LAFOV) PET/CT systems have become available with the conceptual idea to increase system sensitivity and reach a larger anatomical coverage [9]. Current European Association of Nuclear Medicine (EANM) Research Ltd. (EARL) guidelines [5] to support intersystem standardisation of PET imaging, thereby facilitating multicentre studies, have been developed for conventional systems with a 20–25 cm axial field of view (FOV). For LAFOV systems, these performance standards have not yet been adjusted or evaluated.

In order to use our newly installed 106 cm LAFOV Siemens Biograph Vision Quadra PET/CT system (Siemens Healthineers, Knoxville, TN, USA) in multicentre research trials and harmonised clinical use together with conventional FOV systems at our PET centre, compliance to the EARL guidelines needed to be explored. Therefore, in the current study, compliance of the system to EARL standards 1 and 2 throughout the axial FOV was explored to assess whether this system adheres to the European PET image harmonisation guidelines for ^18^F-FDG tumour imaging. Scan duration and/or activity optimization has been explored for the Biograph Vision Quadra PET/CT by Alberts et al. [10]; however, without the use of standardising and harmonising PET image acquisition and reconstruction protocols, they did not focus on maintaining semi-quantitative accuracy. Therefore, clinical data were also collected to further explore and validate the effects of reducing scan duration on semi-quantitative PET image biomarker accuracy and precision for both EARL1 and EARL2 standard compliant reconstruction protocols as well as for clinically optimised reconstruction settings (for maximum ring difference (MRD) = 85 PET data).

## Materials and methods

To test compliance to EARL standards 1 and 2, phantom measurements were performed following EARL standard operating procedure using the NEMA NU2-2001 image quality phantom filled with a sphere-to-background ratio of 10:1 measured in the centre of the FOV [11, 12]. Subsequently, the phantom was placed and measured at various other positions along the axial FOV: one-eighth, a quarter, three-quarters and seven-eighths. Listmode PET data were acquired for 7 min at each position. Subsequently, these data were resampled and reconstructed to represent several shorter scan durations: 60 s, 120 s and 240 s. Images were reconstructed using the three-dimensional (3D) ordered-subset expectation maximisation (OSEM) algorithm with 4 iterations, 5 subsets, matrix size of 220 × 220 × 708 with a voxel size of 3.3 × 3.3 × 1.5 mm^3^, with the application of time of flight (ToF) and resolution modelling (PSF). Different Gaussian filters were applied after reconstruction to comply with EARL standards 1 and/or 2. For each image, the maximum, mean and peak activity concentration recovery coefficients (RC) were derived for all spheres and compared to the EARL standards specifications.

Subsequently, a total of 10 clinically referred oncological patients (6 men, 4 women; age 52–84 years [range], 71 ± 9.0 years [mean ± SD]) received a standard weight-based (3 MBq/kg) intravenous injection of ^18^F-FDG activity (weight: 58–112 kg [range], 79 ± 15 kg [mean ± SD]; activity: 174–305 MBq [range], 238 ± 36 MBq [mean ± SD]), followed by a whole body 10 min listmode PET acquisition. Patients were instructed to fast and avoid exercise at least 4–6 h prior to intravenous ^18^F-FDG injection. Plasma glucose levels were < 8.3 mmol/L before activity administration. PET data were acquired using a single static bed position covering 106 cm (approximately from skull vertex to mid-thigh) at approximately 60 min (± 5%) post-injection. PET data acquired for 10 min were reconstructed, and images at shorter scan times were obtained: 60 s, 120 s, 240 s and 420 s. Images were reconstructed using the vendor-recommended clinically optimised protocol (hereinafter referred to as CLIN) consisting of 3D OSEM with 4 iterations, 5 subsets, a matrix size of 440 × 440 × 708 with a voxel size of 1.6 × 1.6 × 1.5 mm^3^, ToF, PSF and no filtering. In addition, EARL standards 1 and 2 compliant reconstruction settings were used to obtain images adhering to the European guidelines for multicentre PET image quantification and harmonisation which were determined from the phantom measurements described above. For each dataset, tumour lesions were segmented to obtain blood glucose-corrected SUVs using a semi-automated segmentation method (i.e., 50% of SUV_peak_ isocontour). In addition, a 3 cm diameter spherical volume of interest (VOI) was placed in the liver, which served as a reference background uptake VOI and which was used to estimate image noise.

ANOVA repeated measures with post-hoc Bonferroni adjustment for pairwise comparisons were performed to explore differences in lesion SUV_max_ and SUV_peak_ between scan durations in the differently reconstructed images. Differences in liver SUV_peak_ and SUV_mean_ between scan durations in the differently reconstructed images were evaluated by Wilcoxon signed-rank tests. A *P* value of less than 0.05 was considered significant. The coefficient of variation (COV) of the activity concentration (kBq/mL) in the liver VOI was used to characterise noise. Analyses were performed using SPSS Statistics, version 27.0 (IBM corp., Armonk, NY, USA).

Both in the phantom measurements as well as in the patient images, data were acquired using a maximum ring difference (MRD) of 322 crystal rings, while at this time, image reconstructions could only be performed with an MRD of 85 crystals rings [13].

## Results

**Fig. 1:**
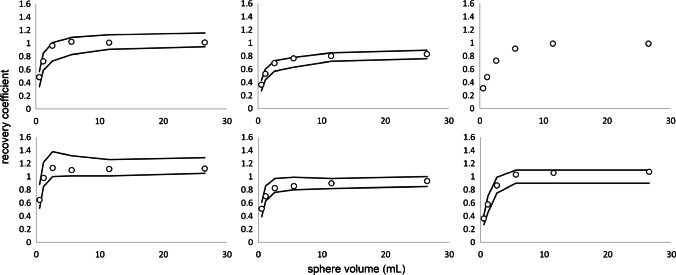
Max (left), mean (middle) and peak (right) RCs as a function of sphere size obtained from images acquired at the centre of the LAFOV reconstructed according to EARL standards 1 (top row) and 2 (bottom row). Solid lines represent the EARL standards acceptability criteria. Please note that EARL 1 standards specifications do not include limits for peak RCs

**Fig. 2:**
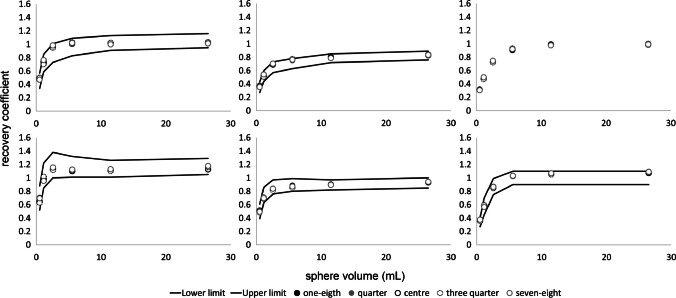
Max (left), mean (middle) and peak (right) RCs as a function of sphere size obtained from images acquired at various positions throughout the LAFOV reconstructed according to EARL standards 1 (top row) and 2 (bottom row). Solid lines represent the EARL standards acceptability criteria. Please note that EARL 1 standards specifications do not include limits for peak RCs

**Fig. 3:**
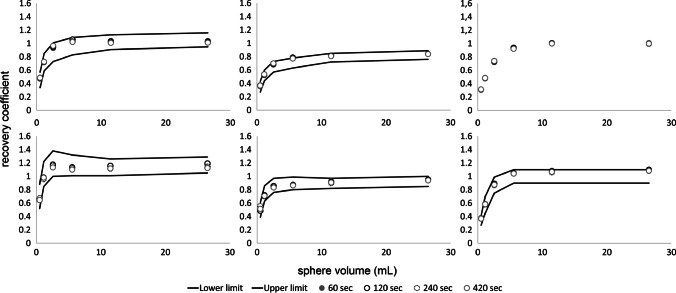
Max (left), mean (middle) and peak (right) RCs as a function of sphere size obtained from images acquired at the centre of the LAFOV reconstructed using various scan durations according to EARL standards 1 (top row) and 2 (bottom row). Solid lines represent the EARL standards acceptability criteria. Please note that EARL 1 standards specifications do not include limits for peak RCs

EARL standards 1 compliance was achieved for all positions along the LAFOV using a 7 mm full width at half maximum (FWHM) Gaussian filter for scan durations of 60 s, 120 s, 240 s and 420 s. However, RCs were close to the specified upper limits. EARL standards 2 compliance was achieved for all positions throughout the LAFOV using a 5 mm FWHM Gaussian filter for all scan durations of 60 s, 120 s, 240 s and 420 s, and all RCs were in the middle between specified upper and lower limits. Figure 1 shows recovery coefficients obtained from images acquired in the centre of the LAFOV for both EARL 1 and 2 standards specifications. For both EARL 1 and 2 standards reconstructions, minimal variability (< 7% for SUV_max_ and < 3% for SUV_peak_ and SUV_mean_) of RCs was observed between the positions tested along the axial FOV. Figure 2 shows recovery coefficients obtained from images acquired at various locations throughout the LAFOV for both EARL 1 and 2 standards specifications. The influence of shorter scan durations on recovery coefficient measurements regarding EARL standards 1 and 2 specifications in the centre of the LAFOV is illustrated in Fig. 3.

**Fig. 4:**
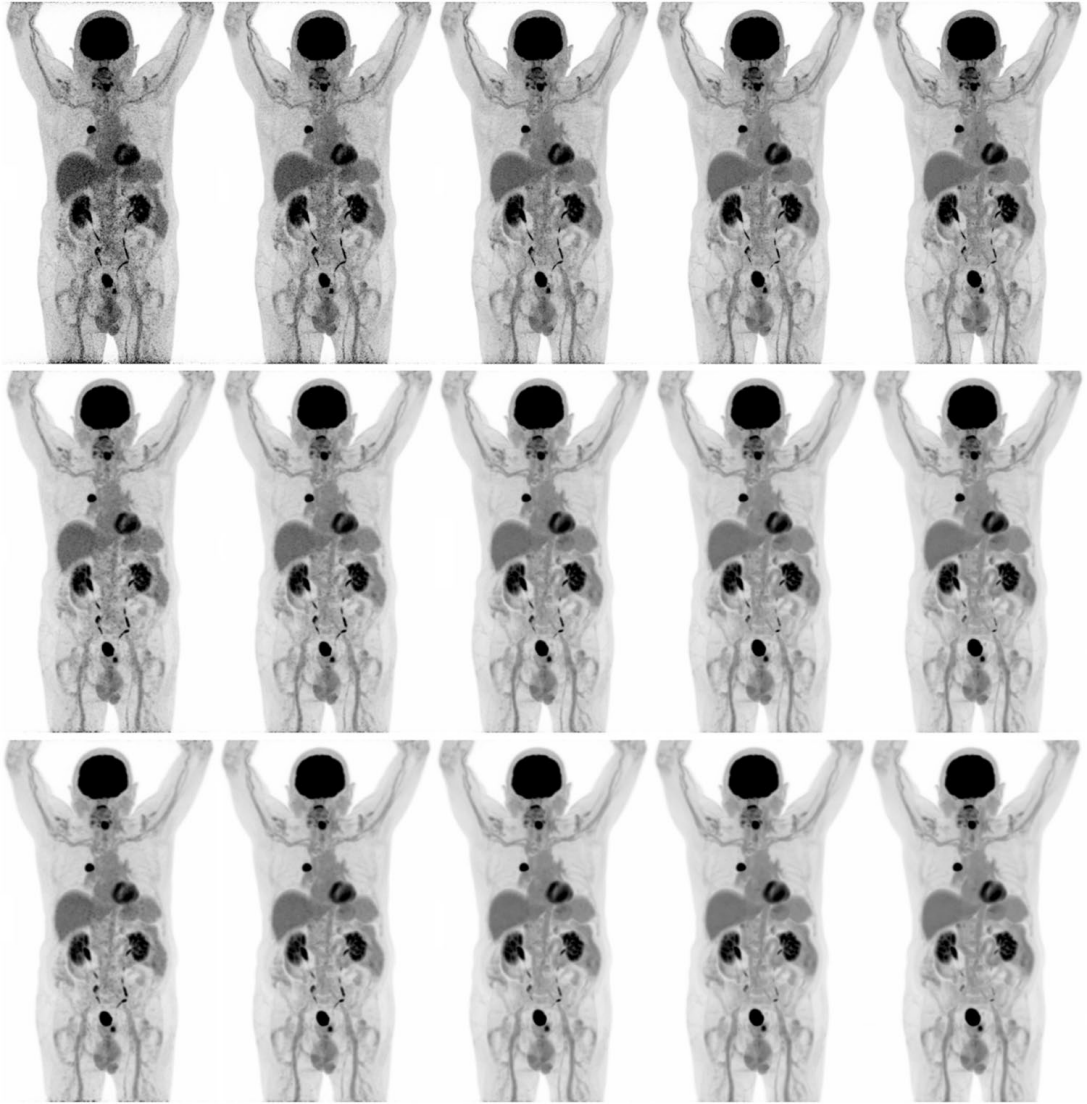
Maximum intensity projection PET images of an 80-year-old male (weight, 93 kg) with right-sided non-small cell lung carcinoma. The patient received a single injection of 279 MBq.^18^F-FDG, and PET images were acquired at 60 min post-injection. Listmode PET data were reconstructed using CLIN (top row), EARL standards 2 (middle row) and EARL standards 1 (bottom row) reconstruction settings for various scan durations: 60 s, 120 s, 240 s, 420 s and 600 s (from left to right)

**Fig. 5:**
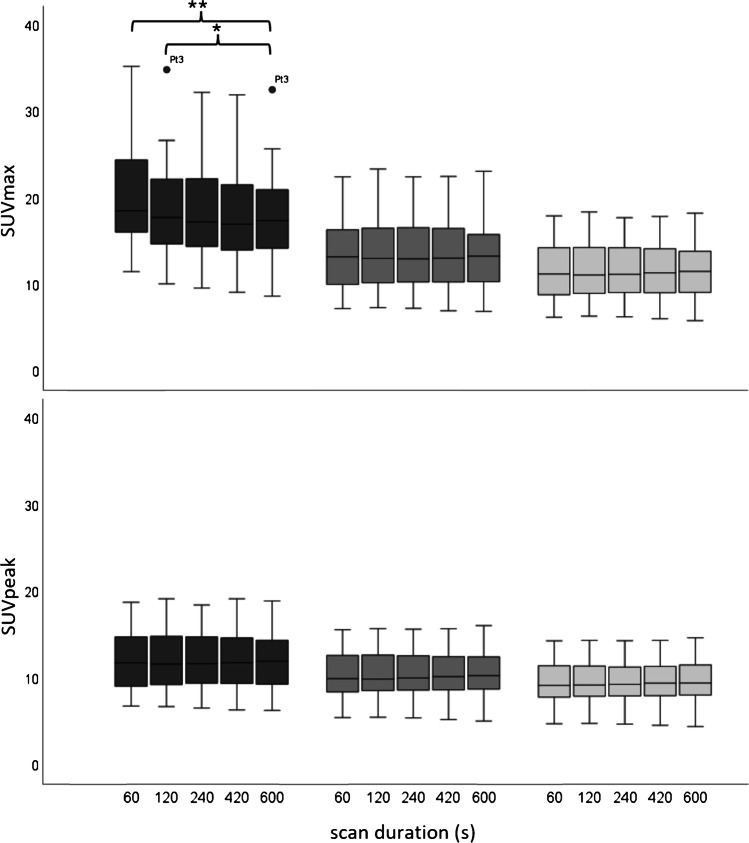
Boxplots illustrating the spread of lesion SUV_max_ (top row) and SUV_peak_ (bottom row) obtained from images reconstructed using the CLIN (dark grey), EARL standards 2 compliant (grey) and EARL standards 1 compliant (light grey) protocol for various scan durations. For a single very ^18^F-FDG avid lesion in one patient, occasionally, some datapoints were seen as an outlier in the boxplot illustrated with circles and labelled with the patient code (Ptx). Single asterisks and double asterisks indicate significant differences between scan durations at *P* < 0.05 and *P* < 0.001, respectively

**Fig. 6:**
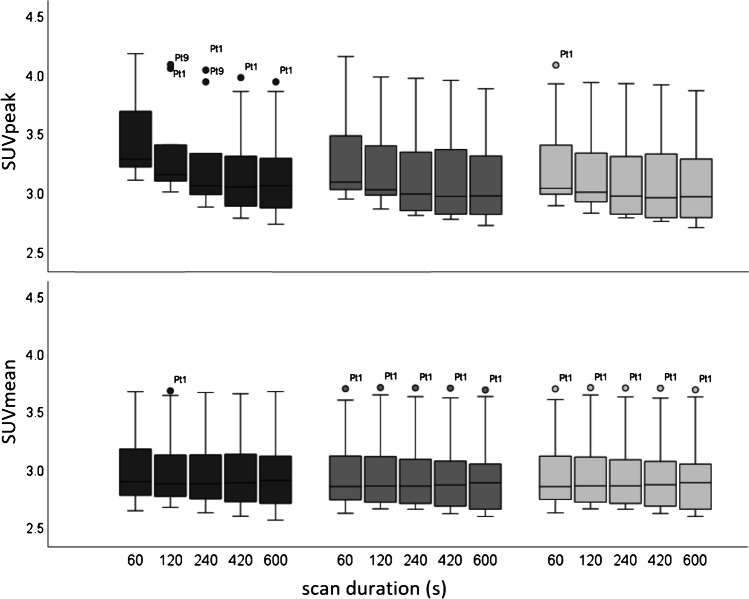
Boxplots illustrating the spread of liver SUV_peak_ (top row) and SUV_mean_ (bottom row) obtained from images reconstructed using the CLIN (dark grey), EARL standards 2 compliant (grey) and EARL standards 1 compliant (light grey) protocol at various scan durations. For one or two subjects with high liver uptake, occasionally, some datapoints were seen as outliers in the boxplot illustrated with circles and labelled with the patient code (Ptx)

Subsequently, a total of 10 oncological patients (6 men, 4 women; age 52–84 years [range], 71 ± 9.0 years [mean ± SD]) received a weight-based (3 MBq/kg) ^18^F-FDG injected activity (weight: 58–112 kg [range], 79 ± 15 kg [mean ± SD]; activity: 174–305 MBq [range], 238 ± 36 MBq [mean ± SD]). Examples of patient maximum intensity projection images at different scan durations, reconstructed according to the CLIN protocol and following EARL standards 1 and 2 reconstruction settings are shown in Fig. 4. Significant differences in lesion SUV_max_ (*n* = 16) were found between the 10 min images and the 60 s (*P* < 0.01; 95% CI, 0.91–3.35) and 120 s (*P* < 0.05; 95% CI, 0.10–2.57) images when reconstructed using the CLIN protocol, whereas no differences were found in lesion SUV_peak_. EARL standards 1 and 2 compliant images did not show significant differences in lesion SUV_max_ and SUV_peak_ between any of the scan durations. An illustration of lesion SUV_max_ and SUV_peak_ obtained from images reconstructed following the CLIN and EARL standards 1 and 2 reconstruction settings at different scan durations is shown in the boxplots of Fig. 5; for an overview of all the corresponding statistical parameters, please refer to Supplementary Table 1. Concerning SUV_peak_ in the liver, significant differences were found in the images obtained using the CLIN reconstruction settings between the 600 s scan duration, and the 420 s (Z-score: − 2.547; *P* value: 0.011), the 240 s (Z-score: − 2.701; *P* value: 0.007), the 120 s (Z-score: − 2.803; *P* value: 0.005) and the 60 s (Z-score: − 2.803; *P* value: 0.005). Furthermore, in the images reconstructed according to EARL standards 1 protocol, a significant difference was found between the 600 s and 240 s images (Z-score: − 2.191; *P* value: 0.028). Regarding SUV_mean_ in the liver, the 600 s images differed significantly from the 60 s images reconstructed according to the CLIN (Z-score: − 2.191; *P* value: 0.028). However, the largest difference in SUV_peak_ was only 0.11 which is clinically a non-relevant difference. For an illustration of the differences in liver SUV_peak_ and SUV_mean_ between scan durations for the differently reconstructed images, see Fig. 6; for an overview of all the corresponding statistical parameters, please refer to Supplementary Table 2.

**Fig. 7:**
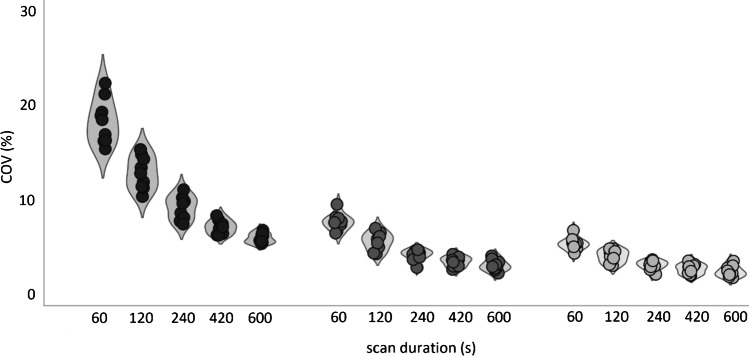
COV of activity concentration (kBq/mL) obtained from a 3 cm diameter spherical VOI placed in a homogeneous part of the liver in images reconstructed using the CLIN (dark grey), EARL standards 2 (grey) and EARL standards 1 (light grey) protocol at various scan durations

Furthermore, the difference in the increase in COV of activity concentration (kBq/mL) in the liver between images obtained using the CLIN and EARL standards 1 and 2 reconstruction settings at different scan durations is illustrated in a violin plot in Fig. 7. COV increased substantially from approximately 7% for the 10 min scan up to 25% with decreasing scan duration for images reconstructed with the CLIN protocol. Unlike images reconstructed according to EARL standards protocols in which COV remained constant from the 10 min to the 240 s scan duration and only increased marginally by approximately 5% from 10 min to 60 s (COV increased from 3 to 8% in images reconstructed using EARL 2 settings).

## Discussion

In the present study, the ability of the Biograph Vision Quadra PET/CT to adhere to the European PET image harmonisation guidelines for ^18^F-FDG tumour imaging, EARL standards 1 and 2, was explored. EARL standards 1 compliance was narrowly achieved as RCs were close to the specified EARL standards 1 limits. The EANM guidelines for tumour imaging version 2.0 [5] describe that spatial filters applied during or after reconstruction should not exceed an FWHM of 7 mm; therefore, further optimization for EARL 1 compliance was not performed. With the emergence of so-called digital PET/CT systems equipped with silicon-based photomultiplier (SiPM) detectors, an update of the guideline was introduced, namely the EARL standards 2 [14, 15]. The Biograph Vision Quadra PET/CT system essentially consists of four interconnected ‘digital’ Biograph Vision PET systems [16] equipped with SiPM-based photon detectors characterised by superior timing resolution, thereby enabling improved ToF estimation and efficient photon detection, a high spatial resolution and, compared to other commercially available PET/CT systems with shorter axial FOV, a higher sensitivity of 16.4 kcps/MBq. These developments in PET technology over the last five years resulting in improved image quality and, thereby, the possibility to reduce either scan duration or amount of administered activity or optimise both, were already impressive [17, 18]. Now, with many more detectors and consequently a substantial increase in sensitivity to 111.5 kcps/MBq (measured using a 140 cm line source in two sets of NEMA sensitivity phantoms (unpublished data, MRD = 85)), the ability of the Biograph Vision Quadra to better adhere to EARL standards 2 than to EARL standards 1 is likely because of improved system performance. As recommended in the literature, the logical next step in clinical practice is to focus solely on EARL 2 standards after finalising the transition period from EARL 1 to EARL 2 [15, 19].

Please note that successive phantom scans were performed at different locations throughout the LAFOV. Data were acquired for 7 min, and no count matching was applied because no impact of increased noise levels was expected. To verify our expectations, the coefficient of variation derived from a 3 mL spherical VOI placed in the background of the unfiltered phantom PET data was calculated and showed an increase from 9 to 13% between the first and last measurement. The results in the current study show that this difference in noise has no substantial or meaningful impact on the observed recovery coefficients as a function of the axial phantom position (variation in recovery was very minimal and would have been even better if count matching would have been applied). Therefore, equivalence has been sufficiently shown as a function of position in the LAFOV.

A portion of the collected clinical data was reconstructed to obtain images at shorter scan durations using different reconstruction protocols, and the effect of scan time reduction on semi-quantitative PET image biomarker accuracy and noise was explored. Here, the reduced scan time can also serve as a surrogate for a reduction in injected activity, with an added 10% to compensate for lower noise-equivalent count rates per MBq at higher activity concentrations [6].

The current study shows that up to a factor of a tenfold reduction in scan duration and/or activity administration is possible when SUV_peak_ is used for semi-quantitative assessment together with EARL-compliant reconstruction settings. This factor of a tenfold reduction in activity administration or scan duration enables new possibilities for research and in clinical settings. A significant reduction in injected activity results in a proportional reduction in radiation exposure, which enables new applications for ^18^F-FDG PET besides tumour imaging. For example, it may become feasible to screen high-risk populations for abnormal cells that may become cancerous in subjects who have no symptoms (yet). In addition, it facilitates PET/CT imaging of children, who are considerably more sensitive to the carcinogenic effects of ionising radiation than adults [20]. On the other hand, a significant reduction in scan duration may make it possible to scan patients who are unable to lay still for a long time, such as intensive care unit (ICU) patients [21], children (without anaesthesia) and patients with severe back pain or claustrophobic patients.

Further research should explore the potential reduction in scan duration and/or activity administration for PET radiotracers with comparatively longer physical half-lives such as ^89^Zr-labelled monoclonal antibodies (mAbs). The slow clearance of mAbs, matching the long physical decay half-life of ^89^Zr, leads to somewhat higher radiation exposure [22]. This limits the amount of activity that can be administered, especially in non-malignant diseases, resulting in poor PET image quality [23]. Hence, at present, long scan durations are required when conventional FOV PET/CT systems are used in order to obtain adequate image quality, especially at later scan time-points post-injection. Consequently, reduced radiation risk may justify the use of ^89^Zr-mAbs in patients with non-malignant diseases too.

Finally, it is important to note that the data acquired in this study were reconstructed using an MRD of only 85 crystal rings, which translates into a photon acceptance angle of 18° [13]. When applying the maximum possible MRD of 322 crystal rings, corresponding to a photon acceptance angle of 52°, the so-called ultra-high sensitivity (UHS) mode becomes available for reconstructing clinical PET data. The sensitivity of the Quadra is rather constant over the LAFOV when using an MRD of 85; when using the UHS mode with an MRD of 322, this is not the case [13]; the peak of the sensitivity is located in the centre of the LAFOV and degrades towards the edges. Changes in sensitivity may induce changes in noise levels which require repetition of the experiments as reported in the current work with additional noise level characterisation using the coefficient of variation. However, using the MRD of 85 represents the lower limit of the system’s potential; the UHS mode will increase image quality even further and will create even more possibilities to reduce scan duration and/or activity administration. Nonetheless, it is expected that the UHS mode will require more careful study due to the fact that the optimal iteration number will need to be explored because of the variant sensitivity and, consequently, noise along the field of view. Furthermore, one may expect a somewhat worse spatial resolution for MRD of 322. These aspects will need to be explored thoroughly in the future, prior to adaptation of the full ring difference for use in clinical routine.

## Conclusion

Images obtained using the LAFOV Biograph Vision Quadra PET/CT (MRD = 85) comply with EARL standards specifications when performing reconstructions using 3D ToF OSEM with 4 iterations and 5 subsets, a matrix size of 220 × 220 × 708, resolution modelling and Gaussian filtering of 7 mm and 5 mm FWHM, respectively. Improved performance characteristics of this LAFOV PET/CT system cause RCs to better fall within lower and upper limits of EARL standards 2 specifications than of EARL standards 1 specifications. Therefore, it is recommended to use EARL standards 2 for image quantification and harmonisation when using the Biograph Vision Quadra PET/CT.

Furthermore, compared to conventional PET/CT systems, scan duration or ^18^F-FDG activity administration could be reduced by a factor of 2.5-fold when SUV_max_ is used combined with CLIN reconstruction settings compared to conventional PET/CT systems. Serious bias in SUV_max_ is induced at shorter scan durations due to increased noise levels (COV increased up to 25%). When images are reconstructed using the protocol defined by EARL standards 2 and semi-quantitative analysis is performed using SUV_peak_, a factor of a tenfold reduction is possible, respectively, without the interference of increased noise (COV increased from 3 to 8%).

## Supplementary Information

Below is the link to the electronic supplementary material.

Supplementary file1 (DOCX 20 KB)

## References

[1] Bastiaannet E, Groen B, Jager PL, Cobben DCP, van der Graaf WTA, Vaalburg W (2004). The value of FDG-PET in the detection, grading and response to therapy of soft tissue and bone sarcomas; a systematic review and meta-analysis. Cancer Treat Rev.

[2] Slomka PJ, Pan T, Germano G (2016). Recent advances and future progress in PET instrumentation. Semin Nucl Med Elsevier.

[3] Hsu DFC, Ilan E, Peterson WT, Uribe J, Lubberink M, Levin CS (2017). Studies of a next-generation silicon-photomultiplier–based time-of-flight PET/CT system. J Nucl Med.

[4] Townsend DW (2008). Dual-modality imaging: combining anatomy and function. J Nucl Med.

[5] Boellaard R, Delgado-Bolton R, Oyen WJG, Giammarile F, Tatsch K, Eschner W (2014). FDG PET/CT EANM procedure guidelines for tumour imaging version 2.0. Eur J Nucl Med Mol Imaging.

[6] Boellaard R, Oyen WJG, Hoekstra CJ, Hoekstra OS, Visser EP, Willemsen AT (2008). The Netherlands protocol for standardisation and quantification of FDG whole body PET studies in multi-centre trials. Eur J Nucl Med Mol Imaging.

[7] Kinahan PE, Fletcher JW (2011). PET/CT standardized uptake values (SUVs) in clinical practice and assessing response to therapy. Semin Ultrasound CT MR.

[8] Wahl RL, Jacene H, Kasamon Y, Lodge MA (2009). From RECIST to PERCIST: evolving considerations for PET response criteria in solid tumors. J Nucl Med.

[9] Vandenberghe S, Moskal P, Karp JS (2020). State of the art in total body PET. EJNMMI Phys EJNMMI Physics.

[10] Alberts I, Hünermund J, Prenosil G, Mingels C, Bohn KP, Viscione M (2021). Clinical performance of long axial field of view PET/CT : a head-to-head intra-individual comparison of the Biograph Vision Quadra with the Biograph Vision PET/CT. Eur J Nucl Med Mol Imaging European Journal of Nuclear Medicine and Molecular Imaging.

[11] Kaalep A, Sera T, Oyen W, Krause BJ, Chiti A, Liu Y (2018). EANM/EARL FDG-PET/CT accreditation – summary results from the first 200 accredited imaging systems. Eur J Nucl Med Mol Imaging.

[12] Boellaard R, Delgado-Bolton R, Oyen WJG, Giammarile F, Tatsch K, Eschner W (2010). FDG PET/CT: EANM procedure guidelines for tumour imaging: version 1.0. Eur J Nucl Med Mol Imaging.

[13] Prenosil GA, Sari H, Fürstner M, Afshar-Oromieh A, Shi K, Rominger A (2021). Performance characteristics of the Biograph Vision Quadra PET/CT system with long axial field of view using the NEMA NU 2–2018 Standard. J Nucl Med.

[14] Boellaard R. New developments of EANM oncology PET/CT guidelines and update of the EARL accreditation standards presentation [Internet]. 2018. Available from: https://eanm-earl-wordpress.esh.netkey.at/wp-content/uploads/2021/04/EARL_18F_stds2_PPP_Boellaard_Oct2018-1.pdf

[15] Boellaard R, Sera T, Kaalep A, Hoekstra OS, Barrington SF, Zijlstra JM (2019). Updating PET/CT performance standards and PET/CT interpretation criteria should go hand in hand. EJNMMI Res EJNMMI Research.

[16] Van SJ, De JJ, Schaar J, Noordzij W, Van SP, Dierckx R (2019). Performance characteristics of the Digital Biograph Vision PET/CT System. J Nucl Med.

[17] van Sluis J, Boellaard R, Somasundaram A, van Snick PH, Borra RJH, Dierckx RAJO (2020). Image quality and semiquantitative measurements on the biograph vision PET/CT system: initial experiences and comparison with the biograph MCT. J Nucl Med.

[18] van Sluis J, Boellaard R, Dierckx RAJO, Stormezand GN, Glaudemans AWJM, Noordzij W (2020). Image quality and activity optimization in oncologic 18F-FDG PET using the Digital Biograph Vision PET/CT System. J Nucl Med.

[19] Kaalep A, Burggraaff CN, Pieplenbosch S, Verwer EE, Sera T, Zijlstra J (2019). Quantitative implications of the updated EARL 2019 PET–CT performance standards. EJNMMI Phys EJNMMI Physics.

[20] Kleinerman RA (2006). Cancer risks following diagnostic and therapeutic radiation exposure in children. Pediatr Radiol.

[21] Pijl JP, Londema M, Kwee TC, Nijsten MWN, Slart RHJA, Dierckx RAJO (2021). FDG-PET/CT in intensive care patients with bloodstream infection. Crit Care.

[22] Bouleau A, Lebon V, Truillet C. PET imaging of immune checkpoint proteins in oncology. Pharmacol. Ther. 2021. 10.1016/j.pharmthera.2020.107786 33307142

[23] Conti M, Eriksson L (2016). Physics of pure and non-pure positron emitters for PET: a review and a discussion. EJNMMI Phys [Internet]. EJNMMI Physics.

