# Supplementary material for: EARL compliance and imaging optimisation on the Biograph Vision Quadra PET/CT using phantom and clinical data
Source: Eur J Nucl Med Mol Imaging. 2022 Jul 25;49(13):4652–60. doi: 10.1007/s00259-022-05919-1 (PMC9606094; doi:10.1007/s00259-022-05919-1)
Supplement: Supplementary file 1 — Supplementary file1 (DOCX 20 KB) [file 259_2022_5919_MOESM1_ESM.docx]

Supplemental Table 1 Differences in lesion SUV_max_ and SUV_peak_ between scan durations of images reconstructed using various reconstruction settings evaluated using ANOVA repeated measures. Single asterisks and double asterisks indicate significant differences between scan durations at *P* < 0.05 and *P* < 0.001, respectively.

| **Semi quantitative parameter** | **Reconstruction settings** | **Scan duration comparison** | **Mean difference** | ***P* value** | **95% confidence interval** |
| --- | --- | --- | --- | --- | --- |
| **Lesion SUV_max_** | CLIN | 600 s – 420 s | -0.168 | 1.000 | -0.792 – 0.456 |
|  |  | 600 s – 240 s | -0.594 | 1.000 | -1.755 – 0.568 |
|  |  | 600 s – 120 s | -1.334 | 0.028* | -2.570 – -0.098 |
|  |  | 600 s – 60 s | -2.132 | 0.000** | -3.351 – -0.913 |
|  | EARL2 | 600 s – 420 s | 0.076 | 1.000 | -0.243 – 0.394 |
|  |  | 600 s – 240 s | 0.085 | 1.000 | -0.313 – 0.483 |
|  |  | 600 s – 120 s | 0.028 | 1.000 | -0.388 – 0.444 |
|  |  | 600 s – 60 s | 0.168 | 1.000 | -0.416 – 0.752 |
|  | EARL1 | 600 s – 420 s | 0.057 | 1.000 | -0.131 – 0.246 |
|  |  | 600 s – 240 s | 0.107 | 1.000 | -0.207 – 0.421 |
|  |  | 600 s – 120 s | 0.036 | 1.000 | -0.269 – 0.341 |
|  |  | 600 s – 60 s | 0.194 | 1.000 | -0.219 – 0.606 |
| **Lesion SUV_peak_** | CLIN | 600 s – 420 s | 0.003 | 1.000 | -0.193 – 0.199 |
|  |  | 600 s – 240 s | 0.074 | 1.000 | -0.224 – 0.372 |
|  |  | 600 s – 120 s | 0.059 | 1.000 | -0.291 – 0.410 |
|  |  | 600 s – 60 s | 0.203 | 1.000 | -0.251 – 0.658 |
|  | EARL2 | 600 s – 420 s | 0.028 | 1.000 | -0.143 – 0.199 |
|  |  | 600 s – 240 s | 0.046 | 1.000 | -0.227 – 0.320 |
|  |  | 600 s – 120 s | 0.053 | 1.000 | -0.223 – 0.329 |
|  |  | 600 s – 60 s | 0.171 | 1.000 | -0.135 – 0.477 |
|  | EARL1 | 600 s – 420 s | 0.036 | 1.000 | -0.103 – 0.175 |
|  |  | 600 s – 240 s | 0.057 | 1.000 | -0.163 – 0.277 |
|  |  | 600 s – 120 s | 0.064 | 1.000 | -0.156 – 0.285 |
|  |  | 600 s – 60 s | 0.140 | 1.000 | -0.118 – 0.398 |

Supplemental Table 2 Differences in background SUV_peak_ and SUV_mean_ between scan durations of images reconstructed using various reconstruction settings evaluated using Wilcoxon signed-rank tests. Single asterisks and double asterisks indicate significant differences between scan durations at *P* < 0.05 and *P* < 0.001, respectively.

| **Semi quantitative parameter** | **Reconstruction settings** | **Scan duration comparison** | **Z-score** | ***P* value** |
| --- | --- | --- | --- | --- |
| **Background SUV_peak_** | CLIN | 600 s – 420 s | -2.547 | 0.011* |
|  |  | 600 s – 240 s | -2.701 | 0.007* |
|  |  | 600 s – 120 s | -2.803 | 0.005* |
|  |  | 600 s – 60 s | -2.803 | 0.005* |
|  | EARL2 | 600 s – 420 s | -1.580 | 0.114 |
|  |  | 600 s – 240 s | -1.784 | 0.074 |
|  |  | 600 s – 120 s | -1.784 | 0.074 |
|  |  | 600 s – 60 s | -1.784 | 0.074 |
|  | EARL1 | 600 s – 420 s | -1.478 | 0.139 |
|  |  | 600 s – 240 s | -2.191 | 0.028* |
|  |  | 600 s – 120 s | -1.784 | 0.074 |
|  |  | 600 s – 60 s | -1.784 | 0.074 |
| **Background SUV_mean_** | CLIN | 600 s – 420 s | -0.357 | 0.721 |
|  |  | 600 s – 240 s | -1.172 | 0.241 |
|  |  | 600 s – 120 s | -1.478 | 0.139 |
|  |  | 600 s – 60 s | -2.191 | 0.028* |
|  | EARL2 | 600 s – 420 s | -1.172 | 0.241 |
|  |  | 600 s – 240 s | -1.274 | 0.203 |
|  |  | 600 s – 120 s | -1.376 | 0.169 |
|  |  | 600 s – 60 s | -0.866 | 0.386 |
|  | EARL1 | 600 s – 420 s | -1.274 | 0.203 |
|  |  | 600 s – 240 s | -1.274 | 0.203 |
|  |  | 600 s – 120 s | -1.376 | 0.169 |
|  |  | 600 s – 60 s | -1.070 | 0.285 |
